# Global Initiative for the Risk Assessment Working Group of Invasive Alien Species

**DOI:** 10.3390/biology14020212

**Published:** 2025-02-18

**Authors:** Xubin Pan

**Affiliations:** Chinese Academy of Inspection and Quarantine, Beijing 100176, China; xubin.hu.pan@gmail.com

Biological invasion is not only an ecological phenomenon but also an administrative challenge. To manage invasive alien species (IAS), given our limited resources, we must be clear about which species, pathways, and habitats should be our top priority, and we can use risk assessment tools to identify and rank them. However, biological invasion risk assessment faces a plethora of dilemmas, which hinders our effective control of IAS. This Special Issue brings together contributions from researchers with different concerns on this topic, and it is hoped that such diverse concerns will attract more attention and motivate us to do more.

The spatial dispersal of IAS means that they cross the boundary of a geographical unit/ecoregion (source area) with these species to reach another geographical unit/ecoregion (sink area) without these species [1]. However, effective management of IAS cannot only consider the sink area, otherwise the source area will provide propagule pressure to the sink area again, resulting in the failure of the IAS control program. Conversely, sink areas can also provide propagule pressure to source areas, as well as other areas, causing more widespread damage. Thus, control measures of IAS must be taken globally.

In fact, international collaboration has become a consensus of IAS management. IAS is listed as an important issue in both the Convention on Biological Diversity agenda and the Sustainable Development Goals. SDSs 15.8 aims to “Prevent Invasive Alien Species on Land and in Water Ecosystems” [2]. However, current international cooperation remains limited to paper to a certain extent, and too much time and resources are wasted in endless meetings. Even Aichi Target 9 states that “By 2020, invasive alien species and pathways are identified and prioritized, priority species are controlled or eradicated, and measures are in place to manage pathways to prevent their introduction and establishment”, which has been only partially achieved [3,4]. Therefore, it is necessary to both rethink and restructure our international efforts to produce concrete results.

Due to the prerequisites and operability of IAS risk assessment, it should become a priority development area for international cooperation [2,3,5]. Here, I propose to initiate a Risk Assessment Working Group of Invasive Alien Species (IAS-RAWG) on a global scale, which can pool the wisdom of global experts and provide imperative support for the targeted management of IAS in countries/areas with insufficient risk assessment capacities.

## 1. Risk Assessment Working Group of Invasive Alien Species

**Figure 1:**
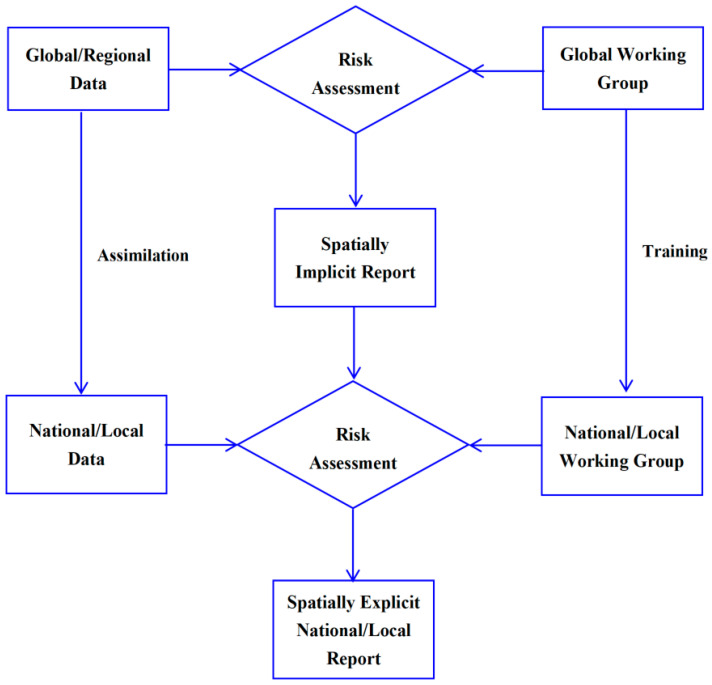
Working flowchart of risk assessment working group of invasive alien species.

The members of IAS-RAWG should come from different countries with various academic backgrounds, and its operating mode can select an iterative mode, such as the two-step method [6]. The global working group uses global/regional data to generate spatial-implicit global reports for risk warning, and the national/local working group then uses the national/local data to generate spatial-explicit national/local reports for risk management (Figure 1). National/local IAS data can be extracted from global/regional data and further added by national/local working groups. At the same time, the global working group can also train national/local working groups to improve their capacities.

## 2. Necessity Analysis of IAS-RAWG

Undoubtedly, risk assessment of IAS is an indispensable component of effective management actions of IAS, through which we can understand which alien species should be concerned. With limited financial resources and unignorable opportunity costs, it is best to give precedence to prevent and control specific species that are likely to cause the most damage.

However, there are still many countries/areas that lack adequate risk assessment capacities, although tremendous progress has been made over the past few decades in identifying IAS and prioritizing them based on the risk they pose, such as the Horizon Scanning Tool (HST) and Pest Risk Analysis (PRA) Tool developed by CABI. Nevertheless, only “invasive alien species are identified and prioritized” in Aichi Target 9 has been or is likely to be achieved, which is almost common sense for IAS, but “priority species are controlled or eradicated” has not been achieved yet, let alone the “measures” [4]. Therefore, it is time to go all out to focus on “priority species” at this stage.

There is a large body of literature focusing on the risk assessment of IAS, exploring not only the pathways and potential geographical distribution of various IAS, but also their impacts and economic losses [7,8]. However, the conclusions from most academic articles remain critically distant from policy and application, as do the recommendations for international cooperation in many places in the discussion section. As we all understand the importance of risk assessment and international cooperation, the global expert community should provide more concrete and practical support.

Risk assessment of IAS is not as simple as it may appear. Widely used methodologies such as SOM and Maxent for the establishment assessment of IAS are not easily learned by beginners through traditional training courses. Meanwhile, risk assessment is not only about techniques but also experiences, which means that learning by ‘doing’ is a better approach for countries or areas with insufficient risk assessment capacities. These countries/areas have little experience in this area, including a lack of experts and organizations, which means they need the fish first and fishing later.

It should be emphasized that the characteristics of risk assessment are particularly suitable for the establishment of a global working group. Global experts can bridge data and knowledge gaps for each other because no one is omnipotent. A reasonable team can have as much information as possible, including not only English, but also Arabic, Chinese, French, Russian, and Spanish. The same is true for assessment techniques, as one person or group cannot master all the methods required. More importantly, group communication can even spark new ideas. Furthermore, this small step in risk assessment with the help of the global group will improve the capacities of countries/areas that are not yet ready for IAS management. In other words, we already have enough “Why” knowledge about IAS, but we need more “How” guidance and even manuals for IAS management in specific countries/areas. 

## 3. Feasibility Analysis of IAS-RAWG

As mentioned above, IAS risk assessment tasks require databases and assessment techniques. There are currently several open-access databases/datasets, including the Global Register of Introduced and Invasive Species, the Global Invasive Species Database, GBIF, CABI-ISC, EPPO Global Database, IPBES IAS Report, InvaCost, Global Naturalized Alien Flora, and so on. Assessment methods include Maxent and Biomod2, SOM, Risk Estimation Matrix [9], EICAT and SEICAT [10,11], and Pest Risk Analysis Standards developed by IPPC (ISPM 2, ISPM 11, and ISPM 21). At the same time, there are many significant findings in the academic literature and reports on the risk assessment of IAS at a global/regional scale, covering the spatial dynamics and the impacts of IAS. In other words, the main gap in the management of IAS exists between the global/regional and national/local scale.

In addition to the database teams and symposium committees, several global expert working groups are currently functioning well. The Global Health Work Group (also known as Epidemiology in Action) focuses on tuberculosis and HIV, and more recently, SARS-CoV-2. IUCN has the Species Survival Commission—Invasive Species Specialist Group. IPPC has established the Phytosanitary Measures Research Group and Beyond Compliance Facilitators. We have also established the International Pest Risk Research Group improving pest risk modeling methods, and another group of 36 experts identifying the drivers of potential alien species impacts [12]. The Mountain Invasion Research Network was established in 2005 to monitor non-native plant invasions in mountainous areas globally. However, it has to be said that compared with international demand and the damages IAS has caused, there are too few global expert working groups specialized in IAS risk assessment. Some countries, such as China and the United States, have indeed taken a similar approach by establishing task forces composed of senior experts from capable national departments and agencies [13].

For countries/areas without IAS management capacities, financial support is a major bottleneck, but for the global risk assessment expert group, this is not a principal problem. On the one hand, most experts should receive financial support from their agencies, international and national funding, or volunteer contributions from IAS assessment recipient countries/areas. If risk assessment groups demonstrate their capacities and contributions, they can apply for further international financial support for more countries/areas or species. On the other hand, the costs for global working groups are negligible, especially in the era of online documents and meetings, which will become the dominant way of working on risk assessment tasks. If working groups need face-to-face discussions on some key issues, they can acquire help from various international symposiums.

## 4. Opportunities and Challenges

IAS management is a systematic project, and risk assessment is an integral part of this work. Risk assessment alone will not prevent or control IAS, and similarly, without risk assessment, IAS management will be ineffective. The successful implementation of a global initiative, therefore, requires that we address the opportunities and challenges that come with it.

### 4.1. Opportunities

Now is the best time for the international community to reach an agreement on this global initiative. Firstly, the IPBES IAS report provides a baseline assessment of IAS, not only including information on status and trends, drivers, impacts, management, and governance and policy options, but also creating various databases. In other words, it is more than just a report; it builds an encyclopedic infrastructure for IAS management, especially risk assessment. Secondly, following Aichi Target 9, the Kunming-Montreal Global Biodiversity Framework provides institutional support in Target 6 to reduce IAS threats to biodiversity [14]. Meanwhile, the WTO/SPS and WHO are also concerned about the global spread of quarantine species. Thirdly, experts in the field of IAS continue to emerge, providing an adequate intelligence pool and academic support. Finally, and most importantly, the development of information technology has provided strong support for IAS management, not only in terms of databases and algorithms, but also through citizen science, online collaboration, and Artificial General Intelligence.

### 4.2. Challenges

However, the implementation of this global initiative faces several challenges. In the first instance, we should take effective actions against IAS as quickly as possible, which will reduce the total cost of damage by the IAS in a specific country/area or at the global/regional scale. In addition, globalization and environmental change will exacerbate the current invasion situation [8]. Next, we should guide national/local governments and experts to conduct a comprehensive IAS survey based on IAS risk assessments, and in turn the background list (IAS catalogue) can become the basis for future IAS risk assessments. Finally, we need to transform our operating mode of IAS management to adapt to the features of IAS management. For risk assessment, it is better to choose a centralized top-down rather than distributed bottom-up approach [15].

## 5. Conclusions

With the global spread and huge impact of IAS, it is time to take effective action as quickly as possible. As risk assessment is important for the management of IAS, I propose a global initiative to establish an international Risk Assessment Working Group of Invasive Alien Species (IAS-RAWG) to overcome the shortcomings of the current discrete assessment mode. The analysis of necessity and feasibility of IAS-RAWG indicates that IAS-RAWG is a useful attempt that is suitable for the development of current technology. Although this mode faces challenges, it also brings valuable opportunities for IAS management.

## Data Availability

Not applicable.

## References

[1] Pan X., Zhang J., Xu H., Zhang X., Zhang W., Song H., Zhu S. (2015). Spatial similarity in the distribution of invasive alien plants and animals in China. Nat. Hazards.

[2] United Nations (2015). Transforming Our World: The 2030 Agenda for Sustainable Development. https://sdgs.un.org/2030agenda.

[3] (2010). Convention on Biological Diversity Aichi Biodiversity Target. https://www.cbd.int/sp/targets.

[4] CBD (2020). Global Biodiversity Outlook 5. https://www.cbd.int/gbo5.

[5] WTO (1994). The WTO Agreement on the Application of Sanitary and Phytosanitary Measures.

[6] Pan X. (2021). Two-step sampling can produce triphasic species-area relationship. J. Plant Ecol..

[7] Diagne C., Leroy B., Gozlan R.E., Vaissière A.C., Assailly C., Nuninger L., Roiz D., Jourdain F., Jarić I., Courchamp F. (2020). InvaCost, a public database of the economic costs of biological invasions worldwide. Sci. Data.

[8] Roy H.E., Pauchard A., Stoett P., Renard Truong T., IPBES (2023). Thematic Assessment Report on Invasive Alien Species and Their Control of the Intergovernmental Science-Policy Platform on Biodiversity and Ecosystem Services.

[9] Department of Agriculture and Water Resources (2016). Biosecurity Import Risk Analysis Guidelines 2016: Managing Biosecurity Risks for Imports into Australia.

[10] Blackburn T.M., Essl F., Evans T., Hulme P.E., Jeschke J.M., Kühn I., Kumschick S., Mrugała A., Marková Z., Nentwig W. (2014). A unified classification of alien taxa based on the magnitude of their environmental impacts. PLoS Biol..

[11] Bacher S., Blackburn T.M., Essl F., Genovesi P., Heikkilä J., Jeschke J.M., Jones G., Keller R., Kenis M., Kueffer C.M. (2018). Socio-economic impact classification of alien taxa (SEICAT). Methods Ecol. Evol..

[12] Essl F., Lenzner B., Bacher S., Bailey S., Capinha C., Daehler C., Dullinger S., Genovesi P., Hui C., Roura-Pascual N. (2020). Drivers of future alien species impacts: An expert-based assessment. Glob. Change Biol..

[13] Herrick C.N. (2019). A review of the U.S. invasive species policy mix: Questioning the prospect of an integrated regime. Environ. Policy Gov..

[14] CBD 15/4. Kunming-Montreal Global Biodiversity Framework. Decision Adopted by the Conference of the Parties to the Convention on Biological Diversity. Proceedings of the Fifteenth Meeting (Part II) of Conference of the Parties to the Convention on Biological Diversity.

[15] Reed M.S., Vella S., Challies E., de Vente J., Frewer L., Hohenwallner-Ries D., Huber T., Neumann R.K., Oughton E.A., Sidoli del Ceno J. (2018). A theory of participation: What makes stakeholder and public engagement in environmental management work?. Restor. Ecol..

